# Novel Highlight in Malarial Drug Discovery: Aspartate Transcarbamoylase

**DOI:** 10.3389/fcimb.2022.841833

**Published:** 2022-03-04

**Authors:** Chao Wang, Arne Krüger, Xiaochen Du, Carsten Wrenger, Matthew R. Groves

**Affiliations:** ^1^ Department of Drug Design, Groningen Research Institute of Pharmacy, University of Groningen, Groningen, Netherlands; ^2^ Unit for Drug Discovery, Department of Parasitology, Institute of Biomedical Sciences, University of São Paulo, São Paulo, Brazil

**Keywords:** aspartate transcarbamoylase, allosteric pocket, pyrimidine biosynthesis, anti-malarials, *Plasmodium falciparum*, X-ray structure

## Abstract

Malaria remains one of the most prominent and dangerous tropical diseases. While artemisinin and analogs have been used as first-line drugs for the past decades, due to the high mutational rate and rapid adaptation to the environment of the parasite, it remains urgent to develop new antimalarials. The pyrimidine biosynthesis pathway plays an important role in cell growth and proliferation. Unlike human host cells, the malarial parasite lacks a functional pyrimidine salvage pathway, meaning that RNA and DNA synthesis is highly dependent on the *de novo* synthesis pathway. Thus, direct or indirect blockage of the pyrimidine biosynthesis pathway can be lethal to the parasite. Aspartate transcarbamoylase (ATCase), catalyzes the second step of the pyrimidine biosynthesis pathway, the condensation of L-aspartate and carbamoyl phosphate to form N-carbamoyl aspartate and inorganic phosphate, and has been demonstrated to be a promising target both for anti-malaria and anti-cancer drug development. This is highlighted by the discovery that at least one of the targets of Torin2 – a potent, yet unselective, antimalarial – is the activity of the parasite transcarbamoylase. Additionally, the recent discovery of an allosteric pocket of the human homology raises the intriguing possibility of species selective ATCase inhibitors. We recently exploited the available crystal structures of the malarial aspartate transcarbamoylase to perform a fragment-based screening to identify hits. In this review, we summarize studies on the structure of *Plasmodium falciparum* ATCase by focusing on an allosteric pocket that supports the catalytic mechanisms.

## Introduction

Malaria is an infectious disease that remains a clear and present threat to human health. It has been estimated that the disease is responsible for more than half a million deaths annually. In 2016, nearly half of the world’s population was at risk of malaria and according to the latest World Malaria Report 2021 (WHO, 2021), there were an estimated 241 million cases of malaria in 2020 in 85 malaria endemic countries, increasing from 227 million in 2019, and malaria deaths increased by 12% compared with 2019, to an estimated 627 000. Children under 5 years accounted for the majority of malaria deaths in areas of high malaria transmission (77% in 2020). To date there are more than 200 known species of the genus *Plasmodium*, but just five of them cause human malaria, comprising *P. vivax*, *P. ovale*, *P. malariae*, *P. knowlesi* and *P. falciparum* (Majori, 2012), the most virulent malaria parasite. A potent vaccine is currently not available and therefore disease control depends mostly on drugs (Greenwood et al., 2008). Malaria is presently undergoing resurgence and the control of *P. falciparum* has become a major challenge in global health. Due to the high mutational rate of the parasite and its resulting rapid adaptation to environmental changes, both drug resistance and the geographic distribution of the disease are increasing (Piel et al., 2010). The inevitable emergence of antimalarial drug resistance (López et al., 2010) forces continuous efforts towards the discovery and development of new antimalarial drugs. There is therefore an urgent need for novel chemotherapeutic targets. Highly attractive avenues for the antimalarial drug discovery are metabolic pathways. The pyrimidine-biosynthesis pathway of *Plasmodium falciparum* is a promising target for antimalarial drug discovery as we reported previously (Lunev et al., 2016; Lunev et al., 2018). Active proliferation during the intraerythrocytic stage of *P. falciparum* requires a supply of purines and pyrimidines for parasite growth to support the production of DNA and parasite replication. As the malarial parasite lacks a purine biosynthetic pathway (De Koning et al., 2005; Hyde, 2007), as well as a functional pyrimidine-import pathway (Reyes et al., 1982; Rathod and Reyes, 1983; Gardner et al., 2002), the parasite relies solely on the *de novo* synthesis pathway to produce pyrimidines, and therefore the *de novo* pyrimidine biosynthesis pathway has been demonstrated to be a promising target for antimalarial drug discovery (Downie et al., 2008; Vaidya and Mather, 2009; Rodrigues et al., 2010; Belen Cassera et al., 2011).

**Figure 1:**
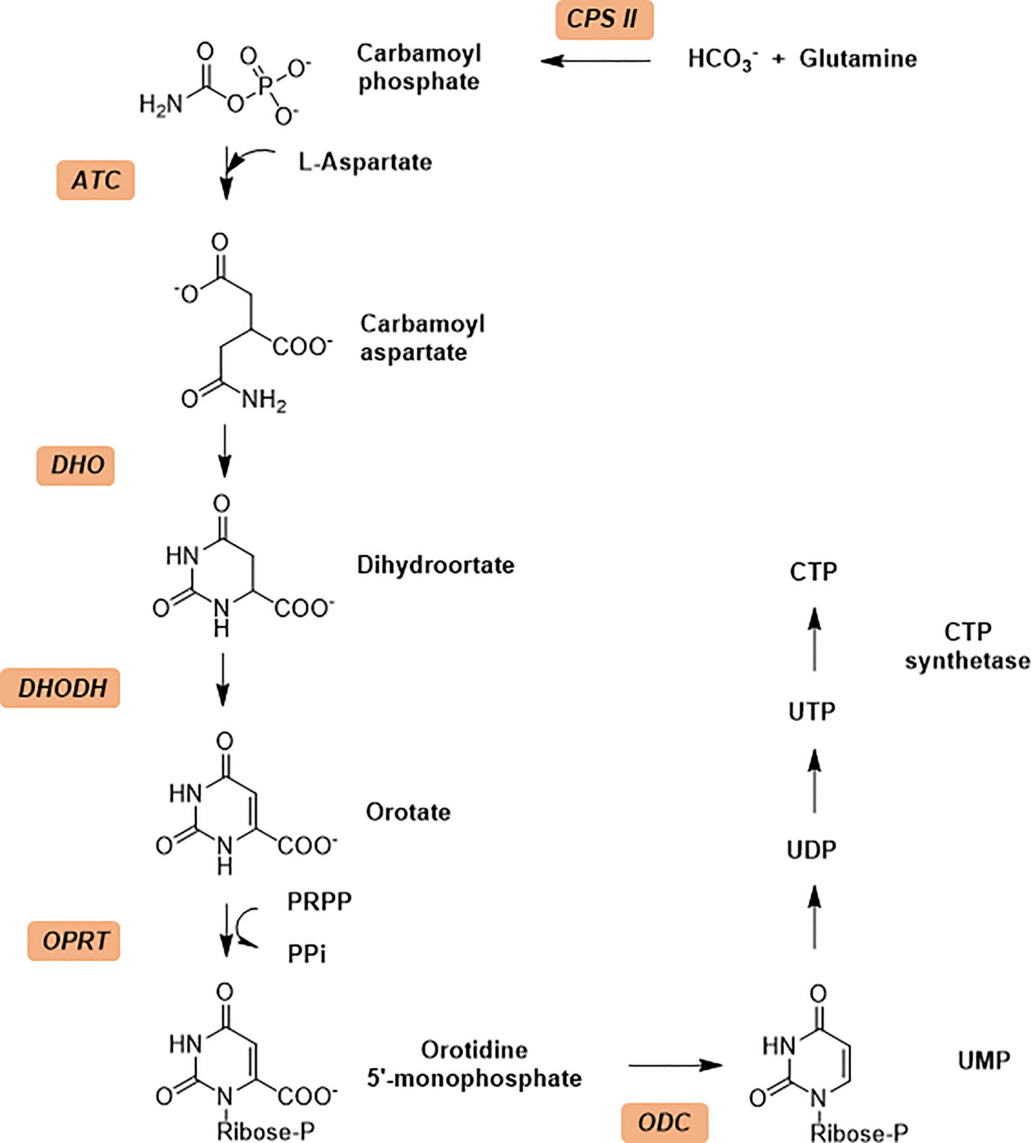
The *de novo* pyrimidine biosynthesis pathway. Enzymes in *de no* pathway are italicized and colored with light orange. Enzymes: CPS II, carbamoyl phosphate synthetase II; ATCase, aspartate transcarbamoylase; DHO, dihydroorotase; DHODH, dihydroorotate dehydrogenase; OPRT, orotate phosphoribosyl transferase; ODC, orotidine 5’-monophosphate decarboxylase.

The *de novo* synthesis of pyrimidines process in general contains six sequential enzymatic steps (**Figure 1**) in *P. falciparum* and starts with the Carbamoyl phosphate synthetase II (CPS II) which is responsible for the formation of carbamoyl phosphate in the cytosol from bicarbonate, glutamine and ATP (Müller et al., 2010; Müller et al., 2010). The Aspartate transcarbamoylase (PF3D7_1344800, ATCase), the second enzyme in the pathway, catalyzes the condensation of aspartate and carbamoyl phosphate to form N-carbamoyl-l-aspartate and inorganic phosphate. The third step is the intramolecular condensation catalyzed by dihyroorotase (DHOase) to the product dihydroorotate. Then dihydroorotate dehydrogenase oxidizes dihydroorotate to orotate. Subsequently orotate and 5-phosphoribosyl-1-pyrophosphate (PRPP) are combined to produce orotidine-5’-monophosphate (OMP) by orotate phosphoribosyl transferase (OPRTase), the final product, molecular uridine monophosphate (UMP), is yielded by decarboxylation of OMP catalyzed by OMP decarboxylase (O’Donovan and Neuhard, 1970; Jones, 1980).

In the following review, we summarize the current knowledge of the *Pf*ATCase, by highlighting the potential of an allosteric pocket and describing how these structures provide insights into the ATCase catalytic mode of action. We will also review current studies on drug discovery against other ATCases, not solely against the human malaria parasite by Torin 2 (Bosch et al., 2020).

## Aspartate Transcarbamoylase Structure and Impact on Mechanism

**Figure 2:**
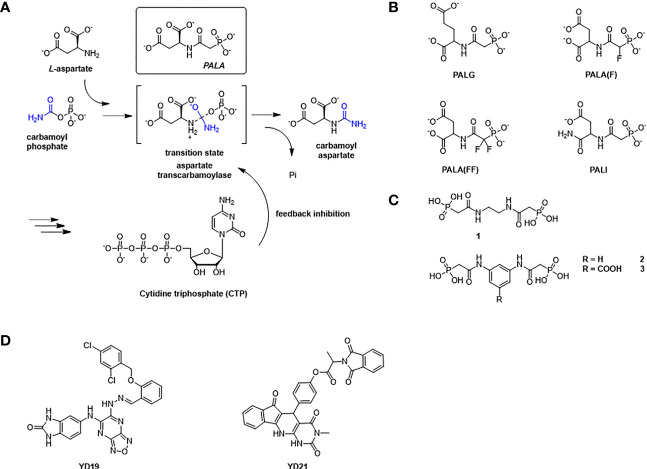
The reaction catalyzed by ATCase and feedback regulation mechanism in *de novo* pyrimidine biosynthesis pathway, and inhibitors against ATCase. **(A)** Aspartate Transcarbamoylase (ATCase) combines L-aspartate and carbamoyl phosphate into carbamoyl aspartate through an enzyme stabilized transition state and inhibition feedback by CTP. The ATC inhibitor PALA closely resembles this transition state intermediate. CTP (a product of the pyrimidine biosynthesis pathway) provides feedback inhibition of ATC activity. **(B)** Structures of PALA analogues as *E. coli* ATCase inhibitors. **(C)** Structures of T-state inhibitors against *E. coli* ATCase that prevent the allosteric transition. **(D)** Structures of allosteric inhibitors of *hu*ATCase *(* Lei et al., 2020).

Aspartate transcarbamoylase catalyzes the second step in *de novo* pyrimidine biosynthesis, where the condensation of L-aspartate (L-Asp) and carbamoyl phosphate (CP) to form N-carbamoyl aspartate (CA) and phosphate (Pi) (**Figure 2A**). The *Escherichia coli* aspartate transcarbamoylase holoenzyme represents the canonical form and is composed of six catalytic and six regulator subunits in which three regulatory pairs coordinate two catalytic trimers (G., 1964; Gerhart and Schachman, 1965; Weber, 1968; Ke et al., 1984; Stevens et al., 1990). The *E. coli* enzyme has been extensively studied and is now a textbook example that regulates pyrimidine biosynthesis pathway through its catalytic and regulatory mechanisms and has been fully characterized by Lipscomb and Kantrowitz (2012). The *E. coli* ATCase is inhibited by the final products of the pyrimidine biosynthesis pathway (CTP) and by a combination of CTP and UTP. The catalytic reaction by the canonical ATCase is sequential (Wang et al., 2005); CP binds first inducing a conformational changes and creating a binding site for L-aspartate. Similarly, N-carbamoyl aspartate leaves the active site before phosphate.

**Figure 3:**
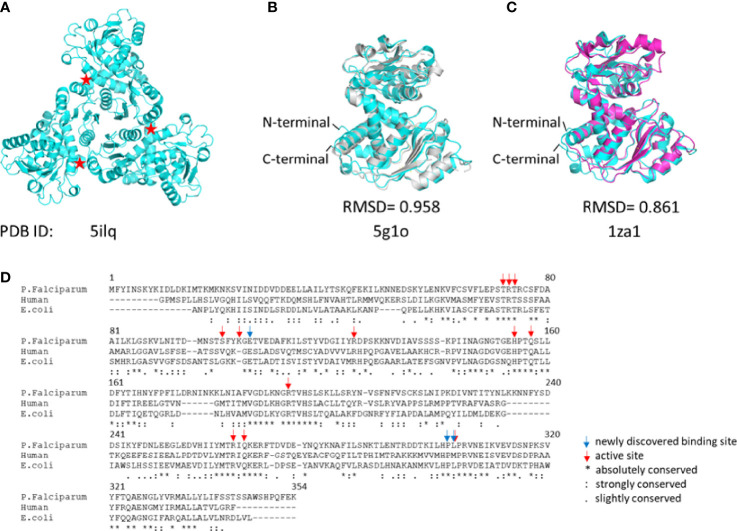
The structure of *Pf*ATCase compare to *hu*ATCase and catalytic subunit of *E.coli* ATCase. **(A)** A ribbon diagram of the crystal structure of the truncated *Pf*ATCase indicating an overall trimeric assembly (Lunev et al., 2016), three active sites formed at the oligomeric interfaces are labeled with stars. **(B)** structural alignment of the monomeric *Pf*ATCase structure (blue, PDB code: 5ILQ) with human ATCase (grey, PDB code: 5G1O) and the catalytic chain of *E.coli* ATCase (magenta, PDB code: 1ZA1) **(C)**. The structural alignments were carried out with Pymol (Schrödinger and DeLano, 2020). **(D)** multiple protein sequence alignment of the human, *P. falciparum* and *E. coli* ATCases using *Tcoffee* (Armougom et al., 2006; Di Tommaso et al., 2011).

The *P. falciparum* ATCase is a homotrimer with three active sites in which each of the three active sites is formed at adjacent oligomeric interface (**Figure 3A**), following the canonical example. Each catalytic site is composed of two functional domains - the aspartate domain, which is mainly responsible for the binding of the substrate L-aspartate, and the carbamoyl phosphate domain, which is mainly responsible for the binding of the substrate carbamoyl phosphate. Apart from the catalytic site, the *E. coli* ATCase also contains regulatory sites, which are targets for the binding of the allosteric effectors – ATP and CTP (Lipscomb and Kantrowitz, 2012). These regulatory sites on the *E. coli* ATCase, are located approximately 60 Å from the closest active site. However, no homologs of the ATCase allosteric chain have been reported in the plasmodial genome.

## Structure of *Plasmodium falciparum* ATCase

*Pf*ATCase is a 43.3 kDa polypeptide with 375 amino acids. In previous studies we determined the crystal structure of truncate aspartate transcarbamoylase from *P. falciparum* (Lunev et al., 2016). Superposition of the *Pf*ATCase structure with catalytic chain of *E. coli* ATCase (PDB code: 1ZA1) and huATCase (PDB code: 5G1O) (**Figures 3B–D**), showed a high degree of homology with the catalytic domain of *E. coli* ATCase and huATCase. Similarly to *E. coli* ATCase, *Pf*ATCase is a homo-trimer in which each of the three active site is formed at the oligomeric interface. Each active site comprises residues from two adjacent subunits in the trimer with a high degree of evolutionary conservation.

## The Structural Changes of ATCase Between T State and R State

As reported the active site of ATCase exists in two distinct states, which are known as the T state and R state. These two states differ in substrate affinity and activity, with the T state, active site present in an open conformation with lower affinity and lower activity for substrate than the R state (Lipscomb and Kantrowitz, 2012; Ruiz-Ramos et al., 2016). We have recently elucidated several structures (Lunev et al., 2016; Lunev et al., 2018) of the plasmodial ATCase (*Pf*ATCase) which provide insight into the conformational changes present in the transition between T and R states in the plasmodial enzyme. While the parasite lacks an ATCase regulatory element, an understanding of this transition may allow for the discovery of drugs that can provide a similar allosteric inhibition impact to that shown by the CTP feedback inhibition of the human and *E. coli* enzymes.

The structures reported by ourselves and others (Lipscomb and Kantrowitz, 2012; Lunev et al., 2016; Ruiz-Ramos et al., 2016) show that the conformation of ATCase can be induced from the T state to the R state when both aspartate and carbamoyl phosphate are present at the active site as well as *N*-phosphonacetyl-L-aspartate (PALA **Figure 2A**) – an analog of the transition state of the reaction catalyzed by ATCase. Additionally, the enzyme can also change from T state to R state by binding carbamoyl phosphate (CP) and succinate, an analog of aspartate.

## Inhibitors Against ATCase

### N-(Phosphonacetyl)-L-Aspartate (PALA)

N-(phosphonacetyl)-L-aspartate (PALA) has been the most potent ATCase inhibitor of ATCase for the past 48 years (Swyryd et al., 1974). PALA is an analog of the transition state of reaction catalyzed by aspartate transcarbamoylase and it combines the structural features of two natural substrates (**Figure 2A**), CP and ASP. The inhibition of PALA against ATCase is competitive with respect to carbamoyl phosphate (CP) and noncompetitive with respect to aspartate (Hoogenraad, 1974; Roberts et al., 1976). From human cell lines and patient tissue samples, PALA was found to bind around 1000 times more tightly than CP, displaying a K_d_ of 0.69 μM (Moore et al., 1982; Baillon et al., 1983). Unfortunately, while PALA is a strong *in vitro* inhibitor of the *E. coli* and human ATC enzymes it is a poor inhibitor of the malarial homolog *in vitro* (Bosch et al., 2020). However, the potential exists that PALA could be developed as an active site inhibitor of the malarial ATCase, as the transition state is identical in all species.

### PALA Analogues

Inspired by success of PALA as a potent inhibitor of ATCase, several groups tried to improve the inhibition ability of PALA for ATCase (**Figure 2B** and **Table 1**). Kafarski et al. (Kafarski et al., 1985) designed a series of phosphate analogues of PALA, as well as the synthesis of other N-(phosphonoacetyl) amino phosphonic acids to evaluate their anticancer activity in human tumor cell lines. However, replacing the α- or β-carboxylic groups in the aspartate moiety by a phosphate group resulted in the total loss of inhibition activity in human KB cell lines. In 2004, Grison et al. (Grison et al., 2004) synthesized N-phosphonoacetyl-L-glutamate (PALG), replacing the aspartate of PALA with glutamate, as the side chain of glutamate is larger than aspartate, they hypothesized that PALG would bind to the open T state and prevent the closure of the two substrate binding domains, thus stabilizing it in the low affinity, low activity T state. The results showed that PALG failed to inhibit *E. coli* ATCase at concentration of 1 mM. Grison et al. also synthesized other analogues of PALA by introducing one [PALA (F)] or two fluorine [PALA (FF)] atoms in the α-position of the phosphorus atom. Unfortunately, PALA(F) inhibited *E.coli* ATCase to only 45% at concentration of 5mM while PALA(FF) show no inhibitory activity towards *E.coli* ATCase. PALA (P) is a bisubstrate analogue, incorporating an element to mimic the leaving phosphate group, that also showed no inhibitory effect on native *E. coli* ATCase. As PALA is highly negatively charged, it is possible that it is difficult for PALA to be transported across lipid bilayers to the enzyme active site (Kempe et al., 1976). Based on this hypothesis, Eldo et al. (Eldo et al., 2006) synthesized the α-amide derivative of PALA, termed PALI, which would reduce the negative charge of the analogue and at the same time enhance the its lipophilicity. The K_d_ of PALI against *E. coli* ATCase was 2 μM, while PALA is reported as 0.69 μM.

**Table 1:** A table summarizing the ATCase inhibitors described in this review.

		Compound code	PDB ID	Activity assay	Compound structure/binding mode
ATCase inhibitors	R state inhibitors	PALA	1D09	*K_i_* = 27 nM (against *E. coli* ATCase)	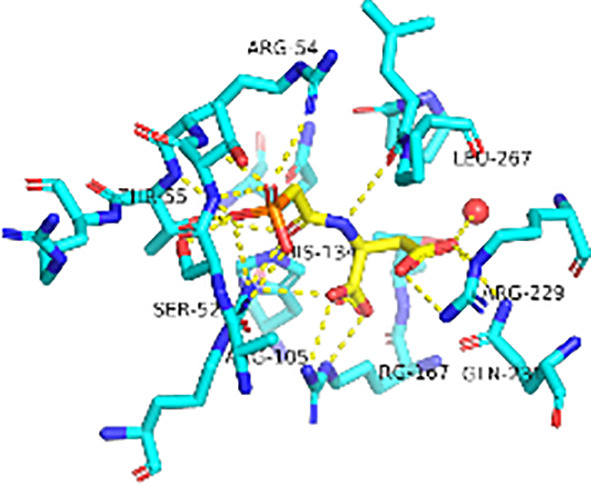
		PALG	*NA*	IC_50_ > 5mM (against *E. coli* ATCase)	*NA*
		PALA(F)	*NA*	IC_50_ > 5mM (against *E. coli* ATCase)	*NA*
		PALA(FF)	*NA*	No inhibition against *E. coli* ATCase	*NA*
		PALI	2H3E	*K_D_* = 2 μM (against *E. coli* ATCase)	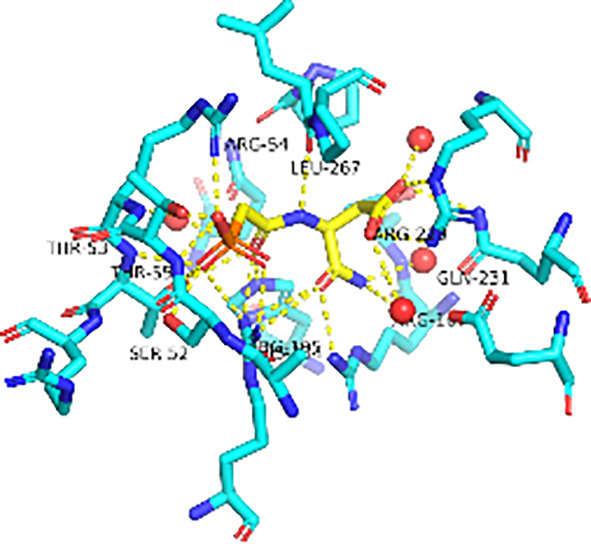
	T state inhibitors	1	2FZC	*K_i_* = 2160 μM (against *E. coli* ATCase)	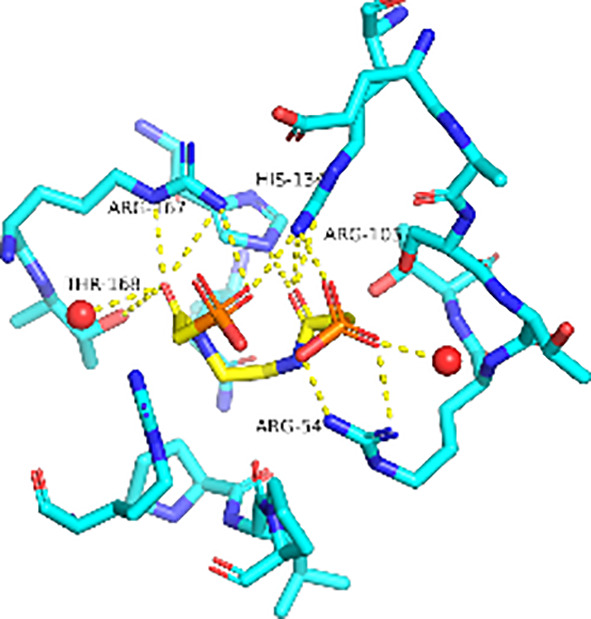
		2	2FZG	*K_i_* = 420 μM (against *E. coli* ATCase)	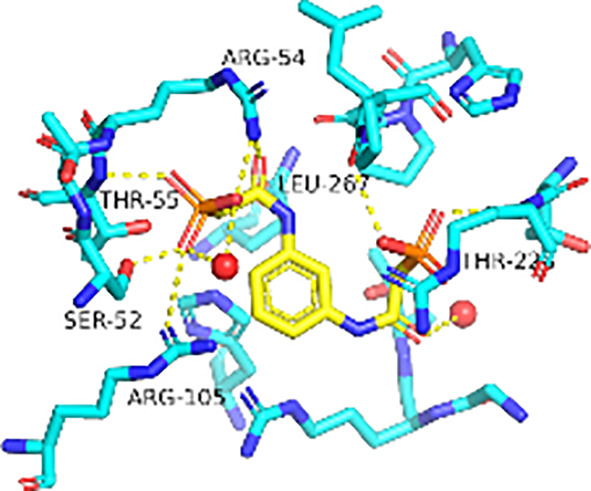
		3	2FZK	*K_i_* = 250 μM (against *E. coli* ATCase)	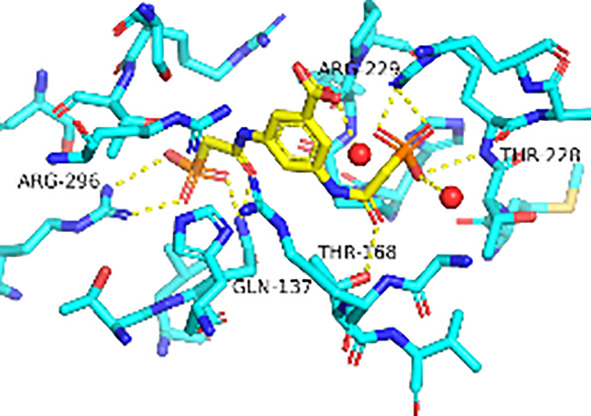
	Allosteric inhibitors	YD19	*NA*	*K_d_* = 12.63 μM (against human ATCase)	*NA*
				*K_d_* = 8.93 μM (against *E. coli* ATCase)	
		YD21	*NA*	*K_d_* = 18.08 μM (against human ATCase)	*NA*
				*K_d_* = 14.06 μM (against *E. coli* ATCase)	

*NA means not available.

### T State ATCase Inhibitors

To generate a class of inhibitors of ATCase targeted at T state of the enzyme, Heng et al. (Heng et al., 2006) synthesized a series of compounds (**Figure 2C**) that were composed of two phosphonacetamide groups linked together based on co-crystal structure of *E. coli* ATCase-CP complex in its T-structure state. The X-ray structure determination of these enzyme-inhibitor complexes showed that these compounds bind to the T state, preventing the conversion of the enzyme to the R state, thereby trapping the enzyme in the low-activity, low affinity T state. However, the K_i_ values for inhibition of ATCase with respect to CP by compounds 1, 2, 3 were 2160, 420, 250 μM, respectively. Compound 1 is a relatively weaker inhibitor compared to compounds 2 and 3.

### Allosteric Inhibitors of Human ATCase

Recently Zhen et al. applied a computational approach to the discovery of inhibitors of the human ATCase (Lei et al., 2020). Based on a large-scale docking experiment the authors identified a series of compounds that were predicted to bind to a previously undetermined allosteric site of the human ATCase. compounds YD19 and YD21 (**Figure 2D**) showed good inhibitory effect on cancer cell lines. Xenograph results showed both YD19 and YD21 inhibited xenograft Hela tumor growth similar to 5FU, one of the most commonly used anti-cancer drugs, as a positive control. These results clearly showed the potential for the discovery of an allosteric pocket for *P. falciparum*.

### Identification of Hits for PfATCase

To identify the initial hits for *Pf*ATCase, we performed a Differential Scanning Fluorimetry (DSF) based screening against our in-house small fragment library (Lunev et al., 2018). 2,3-naphthalenediol showed a significant increase in the thermal stability of *Pf*ATCase, indicating it binds and stabilizes the enzyme *in vitro*. Further, we performed Microscale Thermophoresis (MST) assay to confirm hit binding ability against *Pf*ATCase, with the dissociation constant (Kd) of binding measured as 19.9 ± 4.7 μM. To cross-validate 2, 3-naphthalenediol inhibitory activity against *Pf*ATCase, we performed an enzyme assay *in vitro*, in which 2, 3-naphthalenediol demonstrated inhibition of *Pf*ATCase with an IC_50_ of 5.5 ± 0.9 μM. The X-ray crystal structure of 2, 3-naphthalenediol in complex with *Pf*ATCase was solved at a resolution of 2.0 Å. The *Pf*ATCase-2, 3-naphthalenediol complex crystal structure has been deposited under accession code 6FBA.

## Mechanism of Allosteric Inhibition of *Pf*ATCase by 2,3-Napthalenediol

**Figure 4:**
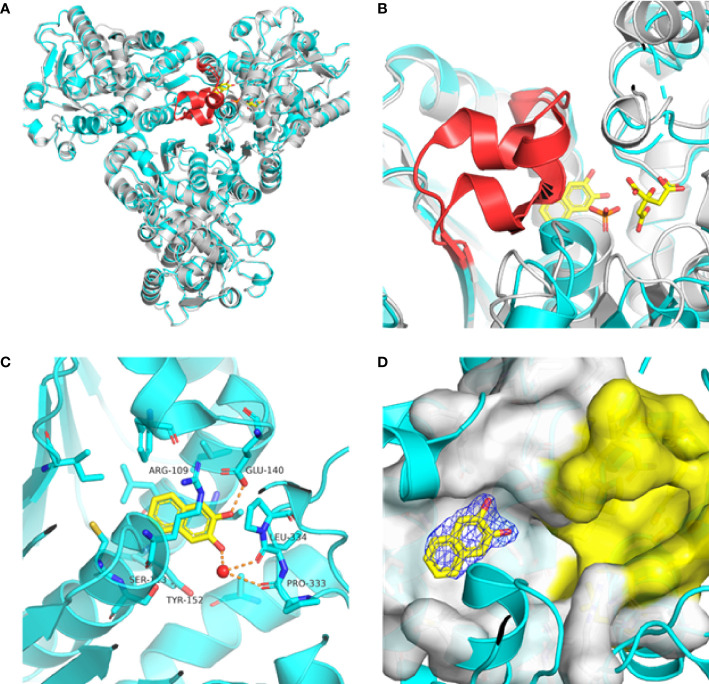
Crystal structure of the 2,3-naphthalenediol-*Pf*ATCase complex. **(A)** structural alignment of the 2,3-naphthalenediol-bounded *Pf*ATCase [PDB ID: 6FBA; blue (Lunev et al., 2018)] with citrate-bound *Pf*ATCase [PDB ID: 5ILN; grey (Lunev et al., 2018)], RMSD=0.478 Å, providing a structural model of *Pf*ATCase in the T-state compared with the R-state. The location of the active site is shown for orientation. The conformational change in the loop128-142 in both cases is highlighted in red. **(B)** shows the structural rearrangements of 2,3-naphthalenediol binding site, the traditional active site is highlighted in yellow for orientation, 2Fo-Fc electron density of 2,3-naphthalenediol is shown in blue mesh at a contour of 1.0σ. **(C)** shows the binding site of 2,3-naphthalenediol and the polar contacts between 2,3-naphthalenediol and surrounding residues. **(D)** magnified view of the newly discovered allosteric binding site and active binding site. The structural alignments were carried out with Pymol (Schrödinger and DeLano, 2020).

Previously, we determined the high-resolution of X-ray crystal structure of the 2, 3-napthalenediol-*Pf*ATCase complex (**Figure 4A**). 2, 3-napthalenediol was identified buried in cavity between two adjacent monomers, which is close to the traditional substrates binding site (**Figure 4B**) and is accommodated in a hydrophobic region buried under the 128-142 loop. Analysis the binding site of 2, 3-napthalenediol reveals the hydroxyl groups of 2, 3-napthalenediol forms polar contact with the side chain of Glu140 and a water bridge with Pro333 and Leu334’s carbonyl main chain oxygens (**Figure 4C**). The structural alignment of the 2, 3-napthalenediol-*Pf*ATCase complex structure with apo-*Pf*ATCase structure did not significantly affect the structure of *Pf*ATCase compared to un-ligand crystal structure. Which strongly suggests that the 2, 3-napthalenediol-*Pf*ATCase complex (PDB ID: 6FBA) is still in T state. In addition, both the X-ray and DSF results determined that 2, 3-napthalenediol can stabilize the *Pf*ATCase.

Furthermore, the comparison of *Pf*ATCase complexed with 2, 3-naphthalenediol, citrate-liganded crystal structures (PDB ID: 5ILN) suggest an allosteric mode of inhibition, as 2, 3-naphthalenediol binds in a cavity between adjacent subunits of the trimer. The 128-142 loop of the citrate-bound *Pf*ATCase loop showed a significant shift compared to 2, 3-naphthalenediol-bound structure (11.2 Å between the alpha-carbons of Thr134 in the two structures) (**Figure 4D**), indicating that 2, 3-naphthalenediol could hold *Pf*ATCase in its low affinity, low activity T-state, preventing the 128-142 loop pushing the Asp domain and CP domain towards each other to form the carbamoyl aspartate and phosphate.

The superposition of the *Pf*ATCase with human ATCase and catalytic subunit of *E.coli* ATCase showed high level of sequence and secondary structure conservation (**Figures 3B–D**). *Tcoffee* (Armougom et al., 2006; Di Tommaso et al., 2011) analysis against human ATCase and the catalytic subunit of *E.coli* ATCase showed that of 12 active site residues 9 (75%) were absolutely conserved, and the residues which we found to have polar contact with 2, 3-naphthalenediol showed 2 of 3 (66.7%) are absolutely conversed.

## Conclusion

Malaria is an infectious disease that remains a clear and present threat to human health. Though several anti-malarial medications are available, the spread of multidrug-resistant severely limits their efficacy. There is pressing need for academic research to discover new targets and drugs for the treatment of severe malaria. For intracellular proliferation, *P. falciparum* requires biosynthesis of pyrimidines for parasite growth to support the production of DNA and parasite replication. ATCase supports the second step of the *de novo* biosynthesis pathway and, as the malaria parasite lacks a functional pyrimidine-import pathway, the *de novo* pyrimidine biosynthesis pathway has been demonstrated to be a major target for antimalarial drug development. While PALA has been long available as a strong ATCase inhibitor, it represents a suboptimal starting point for the development of an anti-malarial for several reasons. Firstly, PALA has been shown to be relatively poor inhibitor of the plasmodial ATCase (Bosch et al., 2020). Secondly, while PALA is a strong *in vitro* inhibitor of canonical ATCases it appears to be limited in development towards a selective inhibitor as it is a mimic of the enzyme transition state. This transition state is likely to be absolutely conserved across species, suggesting that inhibitors of the transition state of the plasmodial homolog may show significant side effects through their inhibition of the human homologs. Finally, the transition state intermediate mimicked by PALA is by nature rather highly charged, which possibly accounts for its relatively poor performance in cell-based assays, as this high charge state is not compatible with efficient transfer across cell membranes. A situation that would be made more challenging when considering the additional membranes that must be traversed to access the parasite ATCase. A potential mechanism to address these limitations and leverage the sensitivity of the parasite to inhibition of *de novo* pyrimidine biosynthesis is to identify allosteric sites of inhibition. Such sites would allow for more selectivity between species and would not be constrained by the limitations imposed by the heavily charged transition state. Our studies have focused on the structure of *Pf*ATCase, and identified an allosteric pocket through the determination of a co-crystal structure of *Pf*ATCase with 2,3-naphthalenediol. By comparing the crystal structure of the 2.3-naphthalenediol:ATCase complex structure as an exemplary of the low-affinity low-activity T-state with a high-affinity high activity R-state represented by the structure of *Pf*ATCase in complex with citrate, there exists the intriguing potential for the development of an allosteric inhibitor of *Pf*ATCase represented by the 2,3-napthalenediol binding site. The further understanding of the mechanism of inhibition of 2,3-napthalenediol would provide an opportunity for further drug development. Such a compound would strengthen the case of *Pf*ATCase as a drug target and would be an invaluable addition to the antimalarial “toolbox”.

## Author Contributions

All authors listed have made a substantial, direct, and intellectual contribution to the work, and approved it for publication.

## Conflict of Interest

The authors declare that the research was conducted in the absence of any commercial or financial relationships that could be construed as a potential conflict of interest.

## Publisher’s Note

All claims expressed in this article are solely those of the authors and do not necessarily represent those of their affiliated organizations, or those of the publisher, the editors and the reviewers. Any product that may be evaluated in this article, or claim that may be made by its manufacturer, is not guaranteed or endorsed by the publisher.
